# Differences in the Effects of Anthocyanin Supplementation on Glucose and Lipid Metabolism According to the Structure of the Main Anthocyanin: A Meta-Analysis of Randomized Controlled Trials

**DOI:** 10.3390/nu13062003

**Published:** 2021-06-10

**Authors:** Risa Araki, Akira Yada, Hirotsugu Ueda, Kenichi Tominaga, Hiroko Isoda

**Affiliations:** 1 Open Innovation Laboratory for Food and Medicinal Resource Engineering (FoodMed-OIL), National Institute of Advanced Industrial Science and Technology (AIST), 1-1-1 Tennodai, Tsukuba 305-8577, Japan; raar51835@gmail.com (R.A.); a-yada@aist.go.jp (A.Y.); ueda.hirotsugu@aist.go.jp (H.U.); k-tominaga@aist.go.jp (K.T.); 2 R&D Center for Tailor-Made QOL, University of Tsukuba, 1-2 Kasuga, Tsukuba 305-8550, Japan; 3 Interdisciplinary Research Center for Catalytic Chemistry, National Institute of Advanced Industrial Science and Technology (AIST), Tsukuba Central 5, 1-1-1 Higashi, Tsukuba 305-8565, Japan; 4 Alliance for Research on the Mediterranean and North Africa (ARENA), University of Tsukuba, 1-1-1 Tennodai, Tsukuba 305-8572, Japan; 5 Faculty of Life and Environmental Sciences, University of Tsukuba, 1-1-1 Tennodai, Tsukuba 305-8572, Japan

**Keywords:** anthocyanins, structure, glucose and lipid metabolism, human health, meta-analysis

## Abstract

The effectiveness of anthocyanins may differ according to their chemical structures; however, randomized clinical controlled trials (RCTs) or meta-analyses that examine the consequences of these structural differences have not been reported yet. In this meta-analysis, anthocyanins in test foods of 18 selected RCTs were categorized into three types: cyanidin-, delphinidin-, and malvidin-based. Delphinidin-based anthocyanins demonstrated significant effects on triglycerides (mean difference (MD): −0.24, *p* < 0.01), low-density lipoprotein cholesterol (LDL-C) (MD: −0.28, *p* < 0.001), and high-density lipoprotein cholesterol (HDL-C) (MD: 0.11, *p* < 0.01), whereas no significant effects were observed for cyanidin- and malvidin-based anthocyanins. Although non-significant, favorable effects on total cholesterol (TC) and HDL-C were observed for cyanidin- and malvidin-based anthocyanins, respectively (both *p* < 0.1). The ascending order of effectiveness on TC and LDL-C was delphinidin-, cyanidin-, and malvidin-based anthocyanins, and the differences among the three groups were significant (both *p* < 0.05). We could not confirm the significant effects of each main anthocyanin on glucose metabolism; however, insulin resistance index changed positively and negatively with cyanidin- and delphinidin-based anthocyanins, respectively. Therefore, foods containing mainly unmethylated anthocyanins, especially with large numbers of OH groups, may improve glucose and lipid metabolism more effectively than those containing methylated anthocyanins.

## 1. Introduction

**Figure 1:**
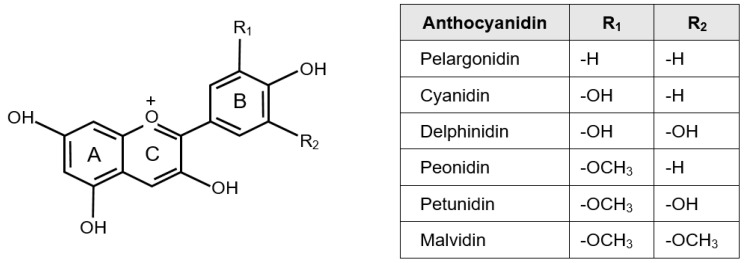
Molecular structures of anthocyanidins.

Anthocyanidins are water-soluble pigments consisting of three ring structures: a double benzoyl ring A and B, and a heterocyclic C ring. They are classified, based on the number of hydroxyl and methoxyl groups attached to the B ring, into six types based: cyanidin, delphinidin, pelargonidin, peonidin, malvidin, and petunidin (Figure 1), the percentage distributions of which in red to purplish-blue-colored foods are 50%, 12%, 12%, 12%, 7%, and 7%, respectively [1,2]. Anthocyanidins are normally present in foods in their glycoside forms, called anthocyanins. Anthocyanins may have organic acids, such as succinic acid and malonic acid, bound to them, apart from sugars, and can be acylated [3]. After absorption, glycosides of cyanidin, delphinidin, pelargonidin, peonidin, malvidin, and petunidin are metabolized to protocatechuic acid (PCA), gallic acid, 4-hydroxybenzoic acid, syringic acid, and vanillic acid, respectively [4].

Of the six compounds, delphinidin has the highest polarity [5], making it more soluble in water than malvidin [6], which has the lowest polarity. Studies have indicated that addition of methoxyl groups to the B ring may reduce solubility [1]. Pelargonidin-based anthocyanins are more easily absorbed than cyanidin-based anthocyanins, and this is suggested to be attributed to the structure of pelargonidin, which cannot undergo methylation due to only one hydroxyl group on B ring and may be more available for glucuronidation [7]. The higher the number of hydroxyl groups, the stronger the blue color of the anthocyanins, and replacing the hydroxyl groups on the B ring with methoxyl groups results in a reddish color. In particular, pelargonidin with 4′-hydroxyl groups, cyanidin with 3′,4′-dihydroxyl groups, and delphinidin with 3′,4′,5′-trihydroxyl groups have orange, reddish-purple, and blue color, respectively [8]. Methoxyl groups in the B ring improve the stability of the anthocyanins in the digestive process, whereas hydroxyl groups reduce it [9]. Moreover, delphinidin-based anthocyanins exhibit higher biophysical interaction than cyanidin- and pelargonidin-based anthocyanins [10].

Anthocyanins are effective antioxidants [11], and their structure, with a positively charged oxygen atom in the C ring [1], lends them their antioxidant activity [12]. Oxidative damage is involved in the onset and progression of various diseases [13], and many health-promoting effects of anthocyanins have been reported in in vitro and clinical studies [1,2,3].

Seeram et al. [14] reported that the antioxidant activities of anthocyanidins increase with the increasing number of hydroxyl groups in the B ring, as evidenced by the following order of decreasing activity: delphinidin with 3′,4′,5′-trihydroxyl groups (70%) > cyanidin with 3′,4′-dihydroxyl groups (60%) > pelargonidin with 4′-hydroxyl groups (40%). They also confirmed a decrease in antioxidant activity on substitution of the hydroxyl groups with methoxyl groups, with the activity in the following order: peonidin with 3′-methoxyl groups (45%) > malvidin with 3′,5′-dimethoxyl groups (43%). Furthermore, the number of glycosyl groups in the A and C rings [15] and the acylation of glycosides [16] have been suggested as factors that reduce the antioxidant activity of anthocyanins.

Excessive intracellular oxidation is considered a risk factor for type 2 diabetes as it leads to inflammation, which in turn induces pancreatic β-cell damage and insulin secretion disorder [17]. Therefore, the differences in the antioxidant activity of anthocyanins owing to their structural differences may variably affect the ability of the pancreas to secrete insulin. Studies have reported a positive association between the ability to secrete insulin and the number of hydroxyl groups in the B rings of anthocyanins [18]. The inhibitory activity of aldose reductase in anthocyanins is attenuated upon methylation of the substituent in the B ring; hence, cyanidin-based anthocyanins show higher activity than peonidin-based anthocyanins [19]. The inhibitory activity of the intestinal and pancreatic glycolytic enzymes increases when glucose is added at the 3-O position but decreases when it is added at the 5-O position [20]. Cell culture studies have reported decreased triglyceride accumulation and mRNA expression of the fatty acid synthase (FAS) and sterol regulatory element-binding protein-1c (SREBP-1c) in 3T3-L1 adipocytes upon treatment with cyanidin compared to that with malvidin [21]. In another experimental study, vascular endothelial growth factor (VEGF) release in vascular smooth muscle cells by platelet-derived growth factor AB (PDGF_AB_) stimulation, one of the risk factors for arteriosclerosis, was inhibited by cyanidin and delphinidin but not by malvidin and peonidin [22]. Additionally, Skemiene et al. has suggested that ischemia-induced activation of the apoptosis-promoting factor caspase depends on the reduction ability of cytosolic cytochrome c, and this ability was the highest in delphinidin-3-glucoside followed by cyanidin-3-glucoside while it was lower in pelargonidin-, malvidin-, and peonidin-3-glucoside [23].

Similarly, the extent to which anthocyanins improve glucose and lipid metabolism among humans may vary depending on their structure and have not yet been investigated using clinical trials or meta-analyses. Therefore, we conducted a meta-analysis of randomized controlled trials (RCTs) to estimate the differences in the effects of anthocyanins on glucose and lipid metabolism based on the structure of the main compound of their sources.

## 2. Materials and Methods

We conducted this meta-analysis based on the Preferred Reporting Items for Systematic Reviews and Meta-Analyses (PRISMA) statement [24].

### 2.1. Search Strategy and Trial Selection

A literature search was conducted up to 5 March 2021, using the databases of PubMed, Cochrane Library, and Web of Science. First, articles were screened according to their titles and abstracts for trials meeting the following inclusion criteria: (1) participants aged ≥18 years; (2) randomized, parallel-group, placebo-controlled clinical trials that compared purified anthocyanins or anthocyanin-rich extracts or anthocyanin-rich food as test foods to placebo or appropriate controls (not therapeutic agents); (3) intervention ≥4 weeks; (4) availability of data for blood triglyceride (TG), total cholesterol (TC), low-density lipoprotein cholesterol (LDL-C), high-density lipoprotein cholesterol (HDL-C), glucose or insulin, or HbA1c levels after fasting or homeostatic model assessment of insulin resistance (HOMA-IR) index; and (5) independent studies written in English.

Trials were excluded if (1) the participants were aged <18 years; (2) a control group was not included or a therapeutic agent was used as a control; (3) anthocyanin supplementation was combined with other functional ingredients; (4) intervention duration was <4 weeks; (5) the intervention was combined with exercise loading; (6) details of the sources of anthocyanins and/or daily dosage of anthocyanins were not described; (7) appropriate data extraction details not provided; (8) the language of publication was not English; or (9) the publication was not an independent research article (e.g., review, conference reports, editorial, and clinical trials registration).

The search terms for PubMed were (“anthocyanin” OR “anthocyanins” OR “cyanidin” OR “delphinidin” OR “malvidin” OR “peonidin” OR “petunidin” OR “pelargonidin”) AND (“randomized clinical controlled trial” OR “randomized controlled trial” OR “RCT”) AND (“metabolic” OR “lipids” OR “TG” OR “triglyceride” OR “TC” OR “LDL” OR “HDL” OR “Glucose” OR “Insulin” OR “HbA1c” OR “HOMA”) NOT (“in vitro” OR “review” OR “bioavailability” OR “kinetics” OR “excretion” OR “children” OR “postprandial” OR “acute”). The search terms for Cochrane Library were (“randomized controlled study” OR “randomized controlled” OR “RCT”) NOT (“review” OR “meta-analysis” OR “postprandial” OR “acute” OR “children” OR “bioavailability” OR “excretion” OR “kinetics” OR “animal” OR in “vitro”) AND (“anthocyanin” OR “anthocyanins” OR “cyanidin” OR “delphinidin” OR “malvidin” OR “peonidin” OR “petunidin” OR “pelargonidin”) AND (“metabolic” OR “cardiovascular” OR “lipids” OR “triglyceride” OR “cholesterol” OR “TC” OR “LDL-C” OR “HDL-C” OR “Glucose” OR “Insulin” OR “HOMA” OR “HbA1c”). The search terms for Web of Science were: #1 (TS = (randomized controlled clinical trial* OR RCT) AND TS = (anthocyanin* OR cyanidin* OR delphinidin* OR malvidin* OR peonidin* OR petunidin* OR pelargonidin) NOT TS = (in vitro* OR review* OR meta-analysis* OR children* OR bioavailability* OR acute* OR postprandial)) AND #2 (TS = (metabolic* OR cardiovascular* OR lipids* OR triglyceride* OR cholesterol* OR LDL* or HDL* OR Glucose* OR insulin* OR HOMA* OR HbA1c* OR TG) AND language: (English) AND document type: (Article)).

**Figure 2:**
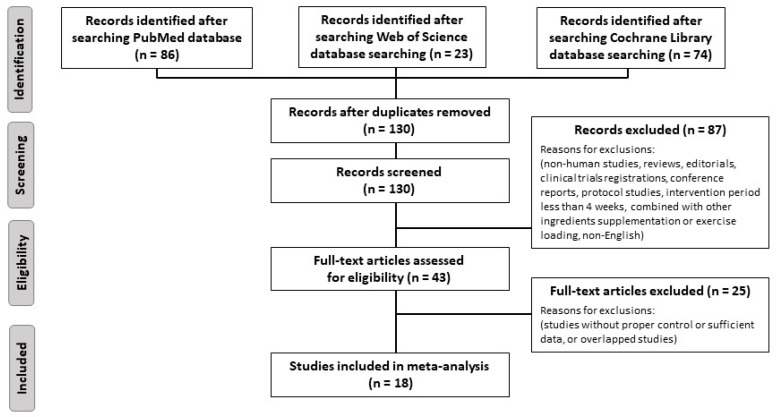
Flow diagram showing the trial selection process.

As shown in Figure 2, a total of 183 articles were obtained in the initial search, of which 130 papers were assessed after removing 53 duplicate articles. Forty-three articles were reviewed in full after reviewing the title and abstract. Of these, we excluded three trials that used the same population as the selected studies. Finally, 18 eligible studies (22 trials) were included in our meta-analysis. 

### 2.2. Outcomes

The primary outcomes of this study were changes in parameters of lipid metabolism (TG, TC, LDL-C, HDL-C) and glucose metabolism (fasting glucose, insulin, HbA1c, HOMA-IR).

### 2.3. Data Extraction and Risk of Bias Assessment

The following information were extracted from each trial: first author, years of data collection, year of publication, clinical characteristics of participants, sample size, duration of intervention, sources of anthocyanins, main anthocyanin in the test foods, daily dosage of anthocyanins, and the means and standard deviations (SD) of glucose and lipid profile at baseline and at the end of the intervention in test and control groups or change by group. If the trial presented the data distribution as standard errors (SE), it was converted to SD by multiplying SE by the square root of the sample size. If the trial presented the data as medians and ranges, it was converted to means and SD using the formula proposed by Hozo et al. [25]. The unit was standardized to mmol/L for TG, TC, LDL, HDL-C, and glucose, µIU/mL for insulin, and %National Glycohemoglobin Standardization Program (NGSP) for HbA1c. 

The anthocyanins in the test foods used in all trials were mixtures of different classes of anthocyanins rather than single compounds. Therefore, we identified the main anthocyanin in the test food as the one mentioned in the articles or its references as the “most abundant” or “predominant”, constituting >50% of the total anthocyanin content, or having the highest content among the six compounds. If such information could not be obtained, we defined the main anthocyanin based on the component composition shown in a related review paper or product information.

We judged the risk of bias of the selected studies as low, high, or unclear according to the Cochrane Handbook for Systematic Reviews of Interventions for the following seven domains: (1) random sequence generation; (2) allocation concealment; (3) blinding of participants and personnel; (4) blinding of outcome assessment; (5) incomplete outcome data; (6) selective reporting; and (7) other potential threats to validity [26]. We assessed the evidence using GRADE criteria (risk of bias; inconsistency of results; indirectness of evidence; imprecision; and publication bias) [27] and created a summary of findings table.

These were conducted by two authors independently, and disagreements were resolved through discussion with all authors.

### 2.4. Data Analysis

This meta-analysis was conducted using RevMan 5.4 (the Cochrane Collaboration, Odense, Denmark) and StatsDirect statistics software (StatsDirect Ltd., Birkenhead, UK). 

The weighted mean differences (MD) for net change and 95% confidence intervals (CI) were used to estimate the effect of anthocyanins on parameters of glucose and lipid metabolism. For trials with ≥2 treatment groups, we compared each treatment group with the control group.

Statistical heterogeneity across studies were assessed using Cochran’s Q test and calculating the *I*^2^ value, and *p* < 0.1 and/or *I*^2^ value ≥ 50 was considered significant [28]. If heterogeneity in either whole food group or each compound group was significant, a random effect model was applied and if none of the heterogeneities were significant, a fixed effects statistical model was applied. To evaluate the robustness of the results and sources of heterogeneity, we performed sensitivity analyses using leave-one-out method. If the significances of the effects on each parameter could not be observed or sensitivity analyses could not be improved for high heterogeneity, subgroup analyses of further subdivisions were performed. Potential publication bias was assessed visually and through Egger’s test [29,30]. Except for heterogeneity, *p* < 0.05 was judged to be statistically significant.

## 3. Results

### 3.1. Study Characteristics

A summary of the characteristics of the 22 trials included in this study are presented in Table 1.

The target population were patients with dyslipidemia in six trials, those with prediabetes and/or type 2 diabetes in three trials, healthy individuals in six trials, overweight or obese individuals in three trials, patients with metabolic syndrome in two trials, and others in two trials. Out of the 22 trials, 20 targeted both genders, and the remaining two trials targeted only men. The source of anthocyanins were various berries, black soybean, and whole purple wheat. The main anthocyanin in the test foods was delphinidin in 13 trials, cyanidin in five trials, and malvidin in four trials. The dosage of anthocyanins ranged from 1.65 to 320 mg/d (mean 160 mg/d), the ratio of main anthocyanin to total anthocyanins ranged from 34% to 98.4%, and the treatment duration ranged from 4 to 24 weeks.

### 3.2. Risk of Bias Assessment

**Figure 3:**
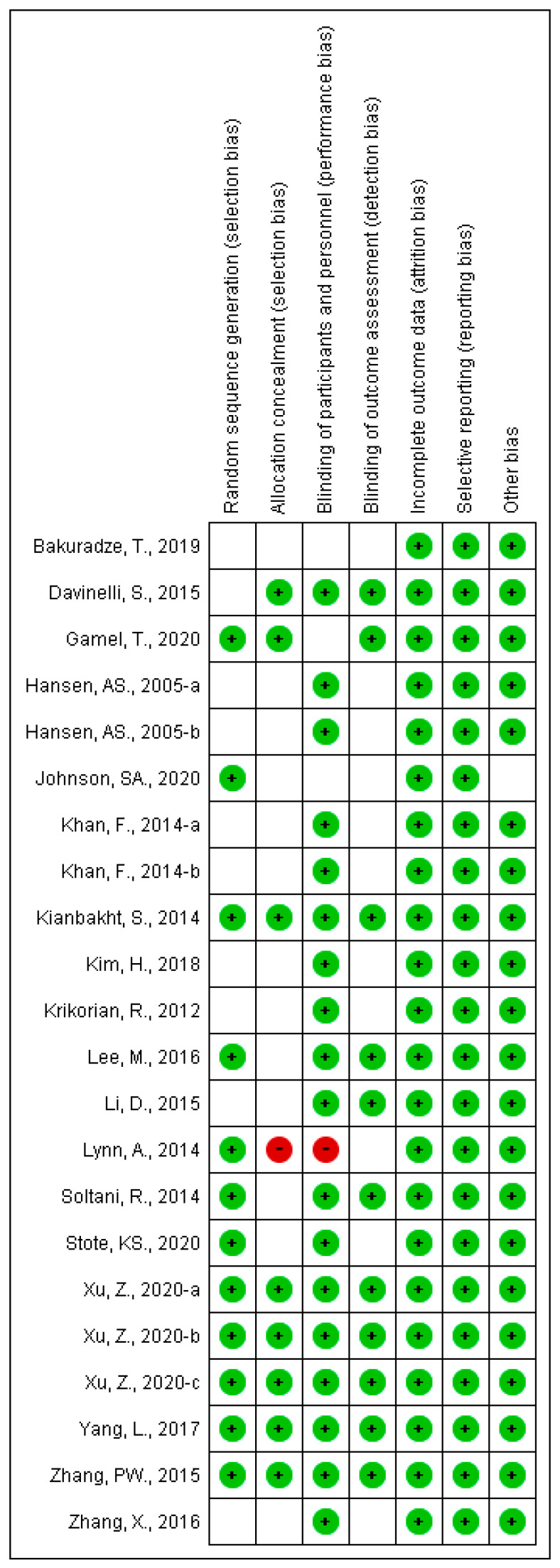
Risk of bias summary. Low risk is indicated as green, high risk as red, and unknown risk as blank.

The results of the risk of bias assessment are shown in Figure 3. Of the 22 selected trials, random sequence generation was conducted in 12 trials. It was confirmed that the allocation concealment was properly performed in eight trials, while the concealment was unclear in 13 trials and was not executed in one trial. Both participants and personnel were blinded, except in three trials (single-blinding in two trials and unblinded in one trial). With respect to incomplete outcome data and selective reporting, all trials were judged to be low-risk. In the trial by Johnson et al. [36], although there was no difference in any outcome, the age at the baseline was significantly different between the test and control groups. Therefore, other risks of bias were evaluated as unclear.

### 3.3. Publication Bias Assessment

Based on Egger’s tests, a significant small-study effect was observed on HDL-C (*p* = 0.004). However, there was no significant evidence of small-study effect on TG (*p* = 0.236), TC (*p* = 0.749), LDL-C (*p* = 0.437), glucose (*p* = 0.962), insulin (*p* = 0.386), HOMA-IR (*p* = 0.825), and HbA1c (*p* = 0.695).

The characteristics of 22 trials (18 studies) are shown. The study by Hansen et al. [35], Khan et al. [37], and Xu et al. [54] had two or more intervention groups. 

### 3.4. Meta-Analysis

#### 3.4.1. Effects of Anthocyanins and Main Anthocyanin in the Test Foods on TG

**Figure 4:**
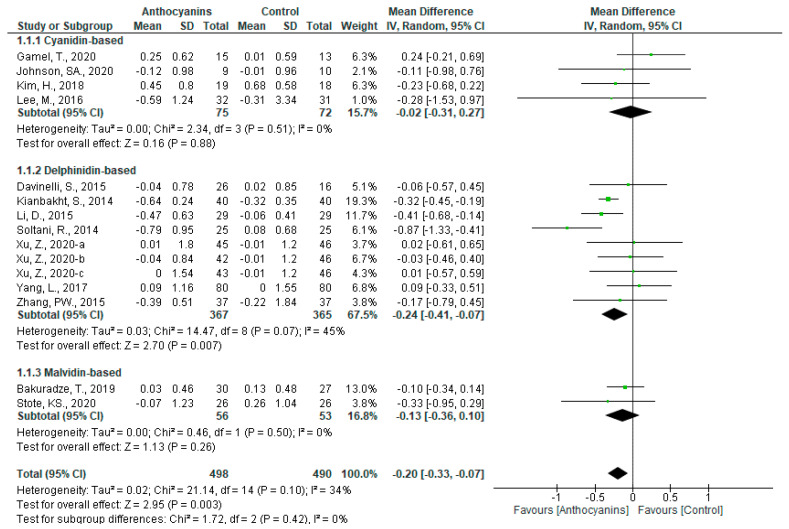
Effects of anthocyanins and main anthocyanin in the test foods on TG.

Favorable effects of anthocyanins were observed (MD: −0.20, 95% CI: −0.33 to −0.07, *p* < 0.01) with moderate heterogeneity (*I*^2^ = 34%, *p* = 0.10), when all the trials that reported TG levels (n = 15) were pooled. When the trials were classified according to the main anthocyanin in the test foods, significant effects were observed only in the trials using delphinidin-based anthocyanins (MD: −0.24, 95% CI: −0.41 to −0.07, *p* < 0.01), with moderate but significant heterogeneity (*I*^2^ = 45%, *p* = 0.07) (Figure 4). 

In the sensitivity analysis, it was observed that the trial by Soltani et al. [50] greatly affected the overall heterogeneity and that of trials using delphinidin-based anthocyanins; after this trial was removed from the dataset, the heterogeneities for all trials and trials using delphinidin-based anthocyanins became non-significant (all trials: *I*^2^ = 4%, *p* = 0.40, trials using delphinidin-based anthocyanins: *I*^2^ = 12%, *p* = 0.34). These overall effects remained significant as shown: all trials (MD: −0.20, 95% CI: −0.30 to −0.11, *p* < 0.0001), and trials using delphinidin-based anthocyanins (MD: −0.23, 95% CI: −0.36 to −0.10, *p* < 0.001). 

The overall effect of anthocyanins was significant in the subgroups “Anthocyanin dosage above 160 mg/d” (MD: −0.18, 95% CI: −0.31 to −0.04, *p* < 0.05), “Anthocyanin extract” (MD: −0.22, 95% CI: −0.43 to −0.02, *p* < 0.05), and “Baseline TG above 1.7 mmol/L” (MD: −0.27, 95% CI: −0.42 to −0.11, *p* < 0.001). Although with significant heterogeneity, the overall effect of trials using delphinidin-based anthocyanins were also significant in the subgroups “Anthocyanin extract” (MD: −0.41, 95% CI: −0.78 to −0.04, *p* < 0.05) and “Baseline TG above 1.7 mmol/L” (MD: −0.25, 95% CI: −0.44 to −0.07, *p* < 0.01). In addition, delphinidin-based anthocyanins were favored in the subgroups “Main anthocyanin to total anthocyanin above 50%” (MD: −0.16, 95% CI: −0.32 to −0.01, *p* = 0.06) with no heterogeneity, and “Dyslipidemia” (MD: −0.27, 95% CI: −0.55 to 0.00, *p* = 0.05) with large heterogeneity (*I*^2^ = 58%, *p* = 0.05) (Table S1).

#### 3.4.2. Effects of Anthocyanins and Main Anthocyanin in the Test Foods on TC

**Figure 5:**
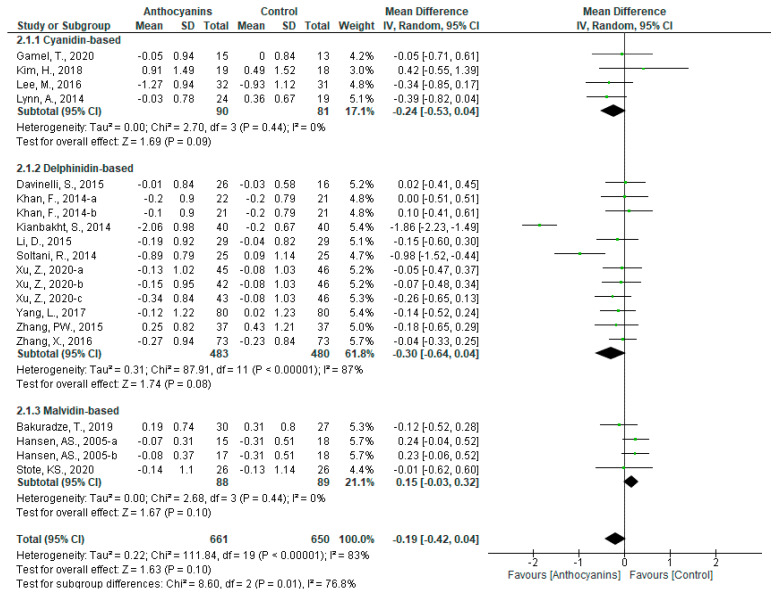
Effects of anthocyanins and main anthocyanin in the test foods on TC.

The effects of anthocyanins on TC levels were not significant (MD: −0.19, 95% CI: −0.42 to 0.04, *p* = 0.10) with very high heterogeneity (*I*^2^ = 83%, *p* < 0.00001) when all the data were pooled (n = 20). Based on the classification of trials according to the main anthocyanin in the test foods, favorable effects were observed in those of cyanidin-based anthocyanins (MD: −0.24, 95% CI: −0.53 to 0.04, *p* = 0.09) with insignificant heterogeneity (*I*^2^ = 0%, *p* = 0.44) and delphinidin-anthocyanins (MD: −0.30, 95% CI: −0.64 to 0.04, *p* = 0.08) with very high heterogeneity (*I*^2^ = 87%, *p* < 0.00001) (Figure 5).

In the sensitivity analyses, it was observed that the trial by Kianbakht et al. [39] highly affected the heterogeneity of all trials and trials using delphinidin-based anthocyanins. After that trial was removed, these heterogeneities became insignificant (all trials: *I*^2^ = 30%, *p* = 0.11, trials of delphinidin-based anthocyanins: *I*^2^ = 16%, *p* = 0.29). The significance of the overall effect of all trials (MD: −0.08, 95% CI: −0.20 to 0.04, *p* = 0.19), and trials using delphinidin-based anthocyanins (MD: −0.14, 95% CI: −0.27 to 0.00, *p* = 0.06) were not changed. 

The results of the subgroup analyses are shown in Table S2. In the subgroup “Main anthocyanin to total anthocyanin above 50%”, the heterogeneity of all trials (*I*^2^ = 0%, *p* = 0.95) and trials using delphinidin-based anthocyanins (*I*^2^ = 0%, *p* = 0.99) reduced to zero. The overall effect of all the trials included became favorable (MD: −0.11, 95% CI: −0.23 to 0.01, *p* = 0.07), whereas the favorable tendency observed for trials using delphinidin-based anthocyanins disappeared (MD: −0.08, 95% CI: −0.21 to 0.05, *p* = 0.21). Similar results were also observed in the subgroup “Anthocyanin dosage above 160 mg/d”. In contrast, in the subgroup “Baseline TC above 5.17 mmol/L”, favorable effects were observed only in the trials using delphinidin-based anthocyanins (MD: −0.40, 95% CI: −0.83 to 0.03, *p* = 0.07) with very high heterogeneity (*I*^2^ = 90%, *p* < 0.00001). Furthermore, in the subgroup “Anthocyanin extract”, overall effects were favorable only according to the pooled data of cyanidin-based anthocyanin trials (MD: −0.24, 95% CI: −0.53 to 0.04, *p* = 0.09). The MD of cyanidin- and delphinidin-based anthocyanin trials showed negative values, while that of malvidin showed positive values. Therefore, a significant difference was observed among the three main anthocyanin trials (*p* < 0.05). 

#### 3.4.3. Effects of Anthocyanins and Main Anthocyanin in the Test Foods on LDL-C 

**Figure 6:**
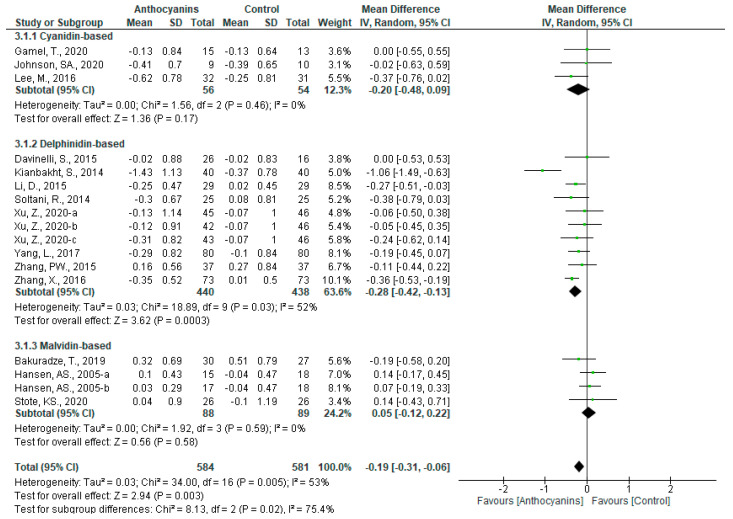
Effects of anthocyanins and main anthocyanin in the test foods on LDL-C.

The favorable effects of anthocyanins on LDL-C levels were significant (MD: −0.19, 95% CI: −0.31 to −0.06, *p* < 0.01) with high heterogeneity (*I*^2^ = 53%, *p* < 0.01) upon pooling data from all trials. When classified according to the main anthocyanin in the test foods, a significant effect was observed only in delphinidin-based anthocyanins (MD: −0.28, 95% CI: −0.42 to −0.13, *p* < 0.001) with large heterogeneity (*I*^2^ = 52%, *p* < 0.05). The differences among the three main anthocyanin groups were also significant (*p* < 0.05) (Figure 6).

In the sensitivity analyses, the trial by Kianbakht et al. [39] affected the heterogeneity of all trials and trials using delphinidin-based anthocyanins. After that trial was removed, heterogeneities of all trials and trials using delphinidin-based anthocyanins became non-significant (all trials: *I*^2^ = 15%, *p* = 0.28; trials using delphinidin-based anthocyanins: *I*^2^ = 0%, *p* = 0.70). These overall effects remained significant in all trials (MD: −0.16, 95% CI: −0.25 to −0.07, *p* < 0.001) and trials using delphinidin-based anthocyanins (MD: −0.25, 95% CI: −0.34 to −0.15, *p* < 0.00001). The significance of the differences among the three main anthocyanin groups was also retained (*p* < 0.05). 

The overall effects of anthocyanins were significant in the subgroups “Main anthocyanin to total anthocyanin above 50%” (MD: −0.24, 95% CI: −0.34 to −0.14, *p* < 0.00001) with no heterogeneity (*I*^2^ = 0%, *p* = 0.71), “Anthocyanin dosage above 160 mg/d” (MD: −0.25, 95% CI: −0.35 to −0.15, *p* < 0.00001) with no heterogeneity (*I*^2^ = 0%, *p* = 0.62), and “Baseline LDL-C levels below 3.6 mmol/L” (MD: −0.16, 95% CI: −0.27 to −0.05, *p* < 0.01) with moderate heterogeneity (*I*^2^ = 30%, *p* = 0.14). The overall effects of delphinidin-based anthocyanins were also significant in the subgroups “Main anthocyanin to total anthocyanin above 50%” (MD: −0.24, 95% CI: −0.34 to −0.14, *p* < 0.00001), “Anthocyanin dosage above 160 mg/d” (MD: −0.26, 95% CI: −0.37 to −0.16, *p* < 0.00001), and “Baseline LDL-C levels below 3.6 mmol/L” (MD: −0.27, 95% CI: −0.38 to 0.17, *p* < 0.00001), none of which showed heterogeneity (all *I*^2^ = 0%, *p* > 0.1). Furthermore, delphinidin-based anthocyanins had significant effects in the subgroups “Purified anthocyanins” (MD: −0.25, 95% CI: −0.35 to −0.15, *p* < 0.00001), “Prediabetes and/or type 2 diabetes” (MD: −0.23, 95% CI: −0.41 to −0.06, *p* < 0.05), “Dyslipidemia” (MD: −0.36, 95% CI: −0.60 to −0.11, *p* < 0.01), and “Baseline BMI above 25.0 kg/m^2^” (MD: −0.29, 95% CI: −0.43 to −0.14, *p* < 0.0001). Except for in the “Dyslipidemia” subgroup (*I*^2^ = 66%, *p* < 0.05), heterogeneity was not significant (all *I*^2^ < 50%, *p* > 0.1) in the other subgroups (Table S3).

#### 3.4.4. Effects of Anthocyanins and Main Anthocyanin in the Test Foods on HDL-C

**Figure 7:**
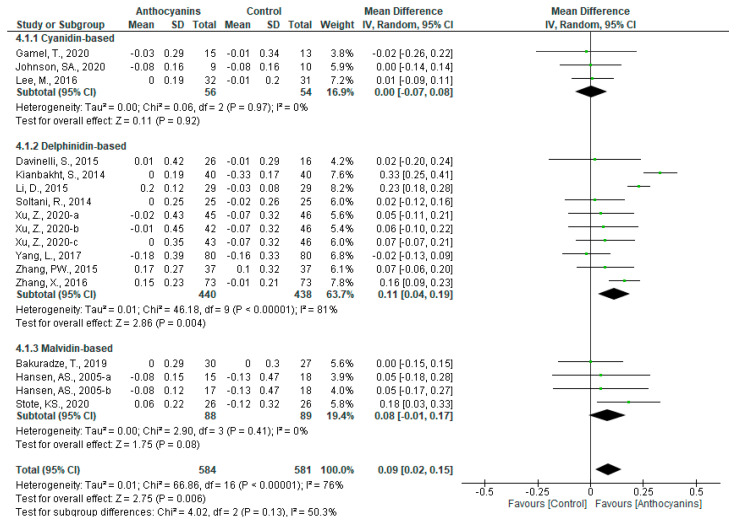
Effects of anthocyanins and main anthocyanin in the test foods on HDL-C.

The favorable effects of anthocyanins on HDL-C levels were significant (MD: 0.09, 95% CI: 0.02 to 0.15, *p* < 0.01) with very high heterogeneity (*I*^2^ = 76%, *p* < 0.00001) when data from all 14 trials were pooled. When classified according to the main anthocyanin in the test foods, significant effects were observed in the trials of delphinidin-based anthocyanins (MD: 0.11, 95% CI: 0.04 to 0.19, *p* < 0.01) with very high heterogeneity (*I*^2^ = 81%, *p* < 0.00001), and favorable effects were observed in the trials of malvidin-based anthocyanins (MD: 0.08, 95% CI: −0.01 to 0.17, *p* = 0.08) with no heterogeneity (*I*^2^ = 0%, *p* = 0.41) (Figure 7). 

In the sensitivity analyses, it was observed that the exclusion of each trial from the dataset did not influence the heterogeneity and overall effect of all trials, as well as that of trials involving cyanidin- and delphinidin-based anthocyanins. However, the overall effect of malvidin-based anthocyanins trials became significant only after removing the trial conducted by Bakuradze et al. [31] (MD: 0.12, 95% CI: 0.01 to 0.23, *p* = 0.03).

In the subgroup “Main anthocyanin to total anthocyanins above 50%”, the heterogeneity of all the trials included and that of trials using delphinidin-based anthocyanins showed a slight decline but remained high, nevertheless, in all trials (*I*^2^ = 73%, *p* < 0.001) and trials using delphinidin-based anthocyanins (*I*^2^ = 72%, *p* < 0.001). Similar results were observed in the following subgroups: “Anthocyanin dosage above 160 mg/d”, “Purified anthocyanin”, and “Baseline HDL-C below 1.4 mmol/L”, while in the subgroup “Baseline BMI above 25.0 kg/m^2^”, all trials (*I*^2^ = 22%, *p* = 0.24) and trials involving delphinidin-based anthocyanins (*I*^2^ = 32%, *p* = 0.22) was insignificant. Additionally, the overall effect in all trials (MD: 0.07, 95% CI: 0.02 to 0.12, *p* < 0.01) and in trials involving delphinidin-based anthocyanins (MD: 0.10, 95% CI: 0.02 to 0.17, *p* < 0.05) remained significant. In this subgroup, the favorable effects of trials involving malvidin-based anthocyanins (MD: 0.12, 95% CI: 0.01 to 0.23, *p* < 0.05) were also significant (Table S4).

#### 3.4.5. Effects of Anthocyanins and Main Anthocyanin in the Test Foods on Glucose

**Figure 8:**
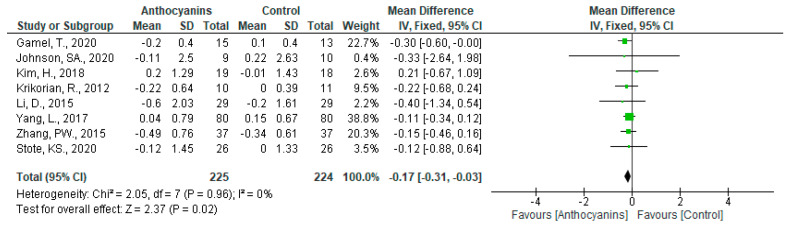
Effects of anthocyanins and main anthocyanin in the test foods on glucose.

The favorable effects of anthocyanins on glucose levels (MD: −0.17, 95% CI: −0.31 to −0.03, *p* < 0.05) without heterogeneity (*I*^2^ = 0%, *p* = 0.96) were observed when all eight trials were pooled (Figure 8). When classified according to the main anthocyanin in the test foods, favorable effects were observed in the trials involving cyanidin-based anthocyanins (MD: −0.25, 95% CI: −0.53 to 0.03, *p* = 0.08) and delphinidin-based anthocyanins (MD: −0.15, 95% CI: −0.31 to 0.02, *p* = 0.09), with no heterogeneity (all *I*^2^ = 0%, *p* > 0.1). 

Because only one trial was applicable, the analysis limited to malvidin-based anthocyanins was omitted.

In sensitivity analyses, the exclusion of each trial did not influence the heterogeneity. However, the overall effect of all trials (MD: −0.13, 95% CI: −0.29 to −0.03, *p* = 0.10) and the trials using cyanidin-based anthocyanins (MD: 0.14, 95% CI: −0.68 to 0.96, *p* = 0.74) became insignificant only after excluding the trial conducted by Gamel et al. [33].

The overall effects of anthocyanins were significant in the subgroups “Main anthocyanin to total anthocyanins above 50%” (MD: −0.17, 95% CI: −0.32 to −0.02, *p* < 0.05), and “Baseline glucose below 7.0 mmol/L” (MD: −0.17, 95% CI: −0.31 to 0.64, *p* < 0.05), none of which showed heterogeneity (all *I*^2^ = 0%, *p* > 0.1). 

#### 3.4.6. Effects of Anthocyanins and Main Anthocyanin in the Test Foods on Insulin

**Figure 9:**
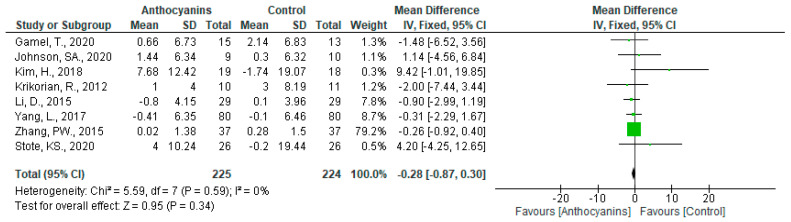
Effects of anthocyanins and main anthocyanin in the test foods on insulin.

The effects of anthocyanins on insulin levels were not significant (MD: −0.28, 95% CI: −0.87 to 0.30, *p* = 0.34), with no heterogeneity (*I*^2^ = 0%, *p* = 0.59) when data from all eight trials were pooled (Figure 9). Based on the classification of trials according to the main anthocyanin in the test foods, both cyanidin-based anthocyanins (MD: 0.80, 95% CI: −2.75 to 4.35, *p* = 0.66) and delphinidin-based anthocyanins (MD: −0.34, 95% CI: −0.93 to 0.26, *p* = 0.27) did not show any significant effects on insulin levels. Because only one trial was applicable, the analysis limited to malvidin-based anthocyanins was omitted.

In the sensitivity analyses, the exclusion of each trial did not influence the heterogeneity and overall effect. No significant effects of anthocyanins or each main anthocyanin on insulin levels were demonstrated through the subgroup analyses (Table S6).

#### 3.4.7. Effects of Anthocyanins and Main Anthocyanin in the Test Foods on HOMA-IR

**Figure 10:**
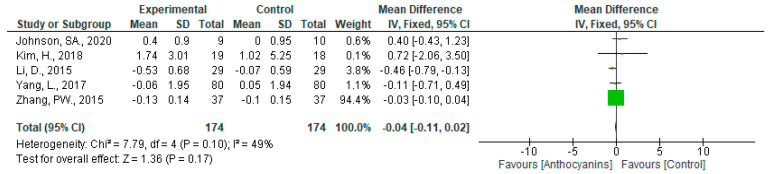
Effects of anthocyanins and main anthocyanin in the test foods on HOMA-IR.

The effects of anthocyanins on HOMA-IR levels were not significant (MD: −0.04, 95% CI: −0.11 to 0.02, *p* = 0.17) with moderate heterogeneity (*I*^2^ = 49%, *p* = 0.10) when data from all eight trials were pooled (Figure 10). 

Although no significant effect was observed in the analyses based on main anthocyanins of the test foods, HOMA-IR changed positively by cyanidin-based anthocyanins (MD: 0.43, 95% CI: −0.37 to 1.22, *p* = 0.29), whereas negatively by delphinidin-based anthocyanins (MD: −0.18, 95% CI: −0.50 to 0.13, *p* = 0.25). Trials using malvidin-based anthocyanins were not included in the HOMA-IR dataset.

In the sensitivity analyses, the exclusion of each trial from the dataset did not influence the heterogeneity and overall effect. 

The overall effect was significant (MD: −0.38, 95% CI: −0.67 to −0.99, *p* < 0.05) with no heterogeneity (*I*^2^ = 0%, *p* = 0.32) in the subgroup “Prediabetes and/or type 2 diabetes” (two delphinidin-based trials). Moreover, similar results were confirmed in the subgroups “Baseline HOMA-IR levels above 2.5” and “Baseline BMI levels below 25.0 kg/m^2^” (Table S7). 

#### 3.4.8. Effects of Anthocyanins and Main Anthocyanin in the Test Foods on HbA1c

**Figure 11:**
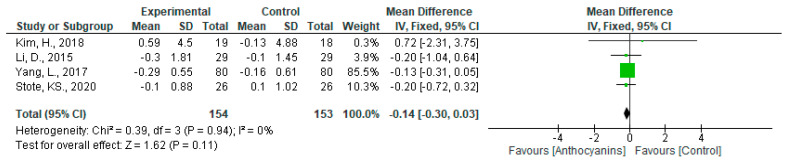
Effects of anthocyanins and main anthocyanin in the test foods on HbA1c.

The effects of anthocyanins on HbA1c levels were not significant (MD: −0.14, 95% CI: −0.30 to 0.03, *p* = 0.11), with no heterogeneity (*I*^2^ = 0%, *p* = 0.94), when data from all five trials were pooled (Figure 11). Moreover, the effect of delphinidin-based anthocyanins was not significant (MD: −0.13, 95% CI: −0.31 to 0.04, *p* = 0.14). Because there was only one trial each using cyanidin- and malvidin-based anthocyanins, analyses of all main anthocyanins except delphinidin-based anthocyanins were omitted.

In the sensitivity analyses, the exclusion of each trial from the dataset did not influence the heterogeneity and overall effect. As shown in Table S8, no subgroup showed a significant trend in terms of overall effect in either all trials or trials using each main anthocyanin.

#### 3.4.9. Summary of Findings

We have summarized our main results in the summary of findings table (Table 2). We judged the quality of the evidence on the outcome of TG, LDL-C, and glucose to be moderate, insulin, HOMA-IR, and HbA1c to be low, and TC and HDL-C to be very low.

## 4. Discussion

In this meta-analysis of 18 RCTs, significant improvements of TG, LDL-C, HDL-C, and glucose by anthocyanins were confirmed. The anthocyanins used in the test foods in these RCTs could be classified into three types: cyanidin-, delphinidin-, and malvidin-based, and overall, the TG, LDL-C, and HDL-C levels were significantly improved only in the trials involving delphinidin-based anthocyanins. The tendency of delphinidin-based anthocyanins to improve TC and glucose levels were also observed. In contrast, the trials involving cyanidin-based anthocyanins only showed improvement in TC and glucose, and malvidin-based anthocyanins only showed improvement in HDL-C. The MD of TC was −0.30, −0.24, and 0.15 for delphinidin-, cyanidin-, and malvidin-based anthocyanins, respectively, and a significant difference was observed among the three groups. The result for LDL-C was similar. Sensitivity analyses indicated that, except for the effects of cyanidin-based anthocyanins on glucose and of malvidin-based anthocyanins on HDL-C, the robustness of the effects of anthocyanins or main anthocyanin in the test foods on glucose and lipid metabolism remains consistent. From these results, the effectiveness of anthocyanins in improving lipid metabolism seemed to be in the following order: delphinidin- > cyanidin- > and malvidin-based. In the present study, it was not possible to examine the extent of the effects of malvidin-based anthocyanins on four parameters related to glucose metabolism and of cyanidin-based anthocyanins on HbA1c because of insufficient data. However, the MD for the four glucose metabolism-related parameters was negative in all trials using delphinidin-based anthocyanins, whereas it was positive in all or some trials using cyanidin-based anthocyanins. Therefore, it was presumed that the effects of each anthocyanin compound on glucose metabolism might be similar to those observed in our meta-analysis of lipid metabolism.

In previous animal studies which confirmed that anthocyanin supplementation decreased fat accumulation and serum lipid levels, the activation of 5′ adenosine monophosphate-activated protein kinase (AMPK) by *Acanthopanax senticosus* [58], suppression of FAS and 3-hydroxy-3-methylglutaryl coenzyme A reductase expression by mulberry water extracts [59], and decreased expression of lipid metabolism-related genes such as peroxisome proliferator-activated receptor γ (PPARγ) and SREBP-1c by *Aronia melanocarpa* extract [60] were observed. Similarly, in cell culture studies, the elevation of phosphorylated AMPK levels [61] and reduction of lipid accumulation and PPARγ protein levels [62] in 3T3-L1 cells by anthocyanins have been reported. Regarding the HDL-C increasing effects of anthocyanins, enhancing cholesterol efflux by activating Liver X receptor α (LXRα) pathway [63] and paraoxonase 1 (PON1) [64] were suggested as related factors. Activation of AMPK by anthocyanins are associated not only with the downregulation of adipogenesis but also the upregulation of glucose transporter type 4 gene expression, glucose uptake elevation [65], and insulin sensitivity improvement [66]. Anthocyanins also act as inhibitors of glycolytic enzymes such as α-glucosidase [67] and sucrase [20]. 

Furthermore, it has been reported that lipid accumulation and mRNA expression of FAS and SREBP1-c in 3T3-L1 adipocytes were decreased when treated with cyanidin rather than malvidin [21], and the inhibitory effects of anthocyanidins on α-glucosidase were in the order of delphinidin > cyanidin > malvidin [68]. In a study by Aboufarrag et al., gallic acid, a delphinidin-3-glucoside metabolite, increased the lactonase activity of PON1 R192R phenotypes significantly, which did not change with PCA, a cyanidin-3-glucoside metabolite, or syringic acid, a malvidin-3-glucoside metabolite [69]. 

These reports suggest that the activity of unmethylated anthocyanins, especially those with large number of OH groups in the B ring, is higher than that of methylated anthocyanins, which supports our results. However, the effects of anthocyanins in this meta-analysis were compared based not on a single compound alone, but on the most abundant anthocyanins in each test food. Therefore, it cannot be denied that the effects observed in this study may have been affected by other compounds coexisting with the main anthocyanins as proved in the animal study by Grace et al. [70]. Moreover, the data of RCTs using pelargonidin-, peonidin-, and petunidin-based anthocyanins were not included in this meta-analysis. The percentages of the main anthocyanin with respect to the total anthocyanins, anthocyanin type, and target population were not completely consistent among trials using cyanidin-, delphinidin-, and malvidin-based anthocyanins. Although we performed subgroup analyses to confirm the effects of confounding factors, they could not be analyzed conclusively due to the small sample size of the selected trials especially for glucose metabolism-related parameters. This may be a reason for the unremarkable effects of anthocyanins on glucose metabolism in our study. In addition, some meta-analyses indicated significant effects of anthocyanins on glucose, and HbA1c [71,72] while no effects of anthocyanins were observed (except for HOMA-IR) in other meta-analyses [73]. Thus, the effects of anthocyanins on glucose metabolism should be judged carefully. Besides, the overall effect of anthocyanins on HDL-C may have a publication bias and low robustness. 

Despite the several limitations mentioned above, to our knowledge, we revealed, for the first time, the possibility that the effects of anthocyanins on glucose and lipid metabolism in humans may be affected by the extent of hydroxylation and methylation of the B ring. In addition, the differences in the effects of the three main anthocyanins were influenced by the type of anthocyanin supplementation and the clinical characteristics of the participants, and it was considered that the conditions under which the differences became remarkable may differ for each parameter. Further studies including RCTs comparing the effects of anthocyanins with different structures will be expected to further the development of personalized medicines.

## 5. Conclusions

In this meta-analysis, it was suggested that foods which contain delphinidin-, cyanidin-, and malvidin-based anthocyanins may be effective for the improvement of lipid metabolism in humans in that order, due to their chemical structure. The differences in the effects of the three main anthocyanins were particularly noticeable for TC and HDL-C. While the effects on the glucose metabolism parameters were not remarkable, they seemed to be more favorable for glucose, insulin, and HOMA-IR in trials using delphinidin-based anthocyanins compared with trials using cyanidin-based anthocyanins. Additionally, it was also suggested that the optimal conditions for achieving the effects of each compound easily, such as the types of anthocyanin source and target population, may differ. The possibility that these findings will contribute to the elucidation of anthocyanin intake to prevent lifestyle-related diseases and atherosclerosis more effectively was indicated.

## Figures and Tables

**Table 1:** Characteristics of selected trials.

Study ID	Participants	Sample Size (n)		Gender	Duration (Week)	Intervention Material	Type of Anthocyanin Source	Dosage (mg/d)	Main Anthocyanins	
		Test Group	Control Group						Compounds [Most Abundant Type]	% of Total ^$^
Bakuradze, T., 2019 [31]	Healthy	30	27	Men	8	Anthocyanin-rich fruit juice	Extract	205.5	malvidin [mv-3-glc]	35.5
Davinelli, S., 2015 [32]	Overweight	26	16	Both	4	Maqui berry extract	Extract	162	delphinidin [unknown]	80
Gamel, T., 2020 [33]	Overweight or obese	15	13	Both	8	Whole purple wheat bars	Extract	1.65	cyanidin [cy-3-glc]	83.7 [34]
Hansen, A.S., 2015-a [35]	Healthy	15	18	Both	4	Red grape extract	Extract	24–36	malvidin [mv-3-glc]	41.2–41.3
Hansen, A.S., 2015-b [35]	Healthy	17	18	Both	4	Red grape extract	Extract	48–71	malvidin [mv-3-glc]	38.1–38.7
Johnson, S.A., 2020 [36]	Metabolic syndrome	9	10	Both	12	Tart cherry juice	Extract	88	cyanidin [cy-3-glc]	42
Khan, F., 2014-a [37]	Healthy	22	21	Both	6	Blackcurrant juice	Extract	40	delphinidin [dp-3-rut]	54.6 [38]
Khan, F., 2014-b [37]	Healthy	21	21	Both	6	Blackcurrant juice	Extract	143	delphinidin [dp-3-rut]	54.6 [38]
Kianbakht, S., 2014 [39]	Dyslipidemia	40	40	Both	8	*Vaccinium arctostaphylos* fruit hydroalcoholic extract	Extract	7.35	delphinidin [unknown]	41.0 [40]
Kim, H., 2018 [41]	Metabolic syndrome	19	18	Both	12	Açaí beverage	Extract	216	cyanidin [cy-3-rut]	98.4 [42]
Krikorian, R., 2012 [43]	Mild cognitive impairment	10	11	Both	16	Concord grape juice	Extract	142–208	delphinidin [dp-3-glc]	40.1 [44]
Lee, M., 2016 [45]	Overweight or obese	32	31	Both	8	Black soybean testa extract	Extract	31.45	cyanidin [cy-3-glc]	68.3
Li, D., 2015 [46]	Type 2 diabetes	29	29	Both	24	Purified anthocyanins from bilberry and blackcurrant	Purified anthocyanins	320	delphinidin [dp-3-glc]	58.0 [47]
Lynn, A., 2014 [48]	Healthy	24	19	Both	6	Tart cherry juice	Extract	273.5	cyanidin [cy-3-rut]	80.0 [49]
Soltani, R., 2014 [50]	Dyslipidemia	25	25	Both	4	*Vaccinium arctostaphylos* L. fruit extract	Extract	90	delphinidin [unknown]	41.0 [51]
Stote, K.S., 2020 [52]	Type 2 diabetes	26	26	Men	8	Freeze-dried blueberries	Others	261.8	malvidin [unknown]	34 [53]
Xu, Z., 2014-a [54]	Dyslipidemia	45	46	Both	12	Purified anthocyanins from bilberry and blackcurrant	Purified anthocyanins	40	delphinidin [dp-3-glc]	58.0 [47]
Xu, Z., 2014-b [54]	Dyslipidemia	42	46	Both	12	Purified anthocyanins from bilberry and blackcurrant	Purified anthocyanins	80	delphinidin [dp-3-glc]	58.0 [47]
Xu, Z., 2014-c [54]	Dyslipidemia	43	46	Both	12	Purified anthocyanins from bilberry and blackcurrant	Purified anthocyanins	320	delphinidin [dp-3-glc]	58.0 [47]
Yang, L., 2017 [55]	Prediabetes and early untreated diabetes	80	80	Both	12	Purified anthocyanins from bilberry and blackcurrant	Purified anthocyanins	320	delphinidin [dp-3-glc]	58.0 [47]
Zhang, P.W., 2015 [56]	NAFLD	37	37	Both	12	Purified anthocyanins from bilberry and blackcurrant	Purified anthocyanins	320	delphinidin [dp-3-glc]	58.0 [47]
Zhang, X., 2016 [57]	Dyslipidemia	73	73	Both	24	Purified anthocyanins from bilberry and blackcurrant	Purified anthocyanins	320	delphinidin [dp-3-glc]	58.0 [47]

The characteristics of 22 trials (18 studies) are shown. The study by Hansen et al. [35], Khan et al. [37], and Xu et al. [54] had two or more intervention groups. ^$^: If the values were not described in the original papers, we applied the values of the references indicated in this column. Cy-3-glc, cyanidin-3-glucoside; dp-3-glc, delphinidin-3-glucoside; mv-3-glc, malvidin-3-glucoside; cy-3-rut, cyanidin-3-rutinoside; dp-3-rut, delphinidin-3-rutinoside.

**Table 2:** Summary of findings for main comparison.

Outcomes	No of Participants (Trials)	Effect Estimates (95% CI)	Quality of the Evidence (GRADE)
TG (mmol/L)	988 (15 trials)	0.20 lower (0.33 lower to 0.07 lower)	㊉㊉㊉◯ Moderate ^a^
TC (mmol/L)	1311 (20 trials)	0.19 lower (0.42 lower to 0.04 higher)	㊉◯◯◯ Very low ^b,c^
LDL-C (mmol/L)	1165 (17 trials)	0.19 lower (0.31 lower to 0.06 lower)	㊉㊉㊉◯ Moderate ^a^
HDL-C (mmol/L)	1165 (17 trials)	0.09 higher (0.02 higher to 0.15 higher)	㊉◯◯◯ Very low ^c,d^
Glucose (mmol/L)	449 (8 trials)	0.17 lower (0.31 lower to 0.03 lower)	㊉㊉㊉◯ Moderate ^b^
Insulin (μU/mL)	449 (8 trials)	0.28 lower (0.87 lower to 0.03 higher)	㊉㊉◯◯ Low ^b,e^
HOMA-IR	348 (5 trials)	0.04 lower (0.11 lower to 0.02 higher)	㊉㊉◯◯ Low ^b,e^
HbA1c (%)	307 (4 trials)	0.14 lower (0.30 lower to 0.03 higher)	㊉㊉◯◯ Low ^b,e^
CI, confidence interval; MD, mean difference; TG, triglyceride; TC, total cholesterol; LDL-C, low-density lipoprotein-cholesterol; HDL-C, high-density lipoprotein-cholesterol; HOMA-IR, homeostatic model assessment of insulin resistance; HbA1c; hemoglobin A1c.			
GRADE Working Group grades of evidence High quality: Further research is very unlikely to change our confidence in the estimate of effect. Moderate quality: Further research is likely to have an important impact on our confidence in the estimate of effect and may change the estimate. Low quality: Further research is very likely to have an important impact on our confidence in the estimate of effect and is likely to change the estimate. Very low quality: We are very uncertain about the estimate.			

^a^: Important inconsistency (substantial heterogeneity (*I*^2^ > 50%)), ^b^: Important imprecision (95% CI close to or crossing the line of no effect), ^c^: Very serious inconsistency (considerable heterogeneity (*I*^2^ > 75%)), ^d^: The possibility of publication bias (Egger’s test was significant (*p* < 0.05)), ^e^: Sparse data (total number of trials < 10).

## Data Availability

Data are available upon the corresponding author after reasonable request.

## Supplementary Materials

The following are available online at https://www.mdpi.com/article/10.3390/nu13062003/s1, Table S1: Subgroup analyses for effects of anthocyanins and main anthocyanin in the test foods on TG, Table S2: Subgroup analyses for effects of anthocyanins and main anthocyanin in the test foods on TC, Table S3: Subgroup analyses for effects of anthocyanins and main anthocyanin in the test foods on LDL-C, Table S4: Subgroup analyses for effects of anthocyanins and main anthocyanin in the test foods on HDL-C, Table S5: Subgroup analyses for effects of anthocyanins and main anthocyanin in the test foods on Glucose, Table S6: Subgroup analyses for effects of anthocyanins and main anthocyanin in the test foods on insulin, Table S7: Subgroup analyses for effects of anthocyanins and main anthocyanin in the test foods on HOMA-IR, Table S8: Subgroup analyses for effects of anthocyanins and main anthocyanin in the test foods on HbA1c.
