# Supplementary material for: Differences in the Effects of Anthocyanin Supplementation on Glucose and Lipid Metabolism According to the Structure of the Main Anthocyanin: A Meta-Analysis of Randomized Controlled Trials
Source: Nutrients. 2021 Jun 10;13(6):2003. doi: 10.3390/nu13062003 (PMC8230537; doi:10.3390/nu13062003)
Supplement: Supplementary file 1 [file nutrients-13-02003-s001.zip › Table S2.pdf]

Table S2. Subgroup analyses for effects of anthocyanins and main anthocyanin in the test foods on TC

Subgroup	Anthocyanin groups	n	MD [95% CI]	I^2	*p* Heterogeneity [a]	*p* Within-group [b]	*p* Intra-group [c]
1. Main anthocyanin to total anthocyanin content							
≥ 50%	Cyanidin-based	4	-0.24 [-0.53, 0.04]	0	0.44	0.09	
	Delphinidin-based	10	-0.08 [-0.21, 0.05]	0	0.99	0.21	
	**Subtotal**	**14**	**-0.11 [-0.23, 0.01]**	**0**	**0.95**	**0.07**	**0.31**
< 50%	Delphinidin-based	2	-1.44 [-2.30, -0.58]	86	0.009	0.001	
	Malvidin-based	4	0.15 [-0.03, 0.32]	0	0.44	0.10	
	**Subtotal**	**6**	**-0.41 [-1.12, 0.30]**	**95**	**< 0.00001**	**0.25**	**0.0004**
2. Anthocyanin dosage							
≥ 160 mg	Cyanidin-based	2	-0.11 [-0.86, 0.65]	55	0.14	0.78	
	Delphinidin-based	6	-0.11 [-0.27, 0.04]	0	0.94	0.15	
	Malvidin-based	2	-0.09 [-0.42, 0.25]	0	0.77	0.61	
	**Subtotal**	**10**	**-0.13 [-0.26, 0.01]**	**0**	**0.91**	**0.07**	**0.99**
< 160 mg	Cyanidin-based	2	-0.23 [-0.64, 0.17]	0	0.50	0.26	
	Delphinidin-based	6	-0.48 [-1.18, 0.22]	93	< 0.00001	0.18	
	Malvidin-based	2	0.24 [0.03, 0.44]	0	0.96	0.02	
	**Subtotal**	**10**	**-0.28 [-0.73, 0.18]**	**92**	**< 0.00001**	**0.23**	**0.03**
3. Types of anthocyanin source							
Purified anthocyanins	Delphinidin-based	7	-0.12 [-0.26, 0.03]	0	0.98	0.12	
	**Subtotal**	**7**	**-0.12 [-0.26, 0.03]**	**0**	**0.98**	**0.12**	**-**
Extract	Cyanidin-based	4	-0.24 [-0.53, 0.04]	0	0.44	0.09	
	Delphinidin-based	5	-0.55 [-1.40, 0.30]	94	< 0.00001	0.20	
	Malvidin-based	3	0.16 [-0.04, 0.36]	20	0.30	0.13	
	**Subtotal**	**12**	**-0.24 [-0.64, 0.16]**	**90**	**< 0.00001**	**0.23**	**0.03**
4. Target population							
Prediabetes and/or type 2 diabetes	Delphinidin-based	2	-0.14 [-0.43, 0.15]	0	0.97	0.33	
	**Subtotal**	**3**	**-0.12 [-0.38, 0.14]**	**0**	**0.93**	**0.37**	**0.70**
Dyslipidemia	Delphinidin-based	6	-0.57 [-1.20, 0.07]	92	< 0.00001	0.08	
	**Subtotal**	**6**	**-0.57 [-1.20, 0.07]**	**92**	**< 0.00001**	**0.08**	**-**
Overweight or obesity	Cyanidin-based	2	-0.23 [-0.62, 0.17]	0	0.50	0.26	
	**Subtotal**	**3**	**-0.11 [-0.41, 0.18]**	**0**	**0.56**	**0.45**	**0.45**
Healthy	Delphinidin-based	2	0.05 [-0.31, 0.41]	0	0.79	0.79	

	Malvidin-based	3	0.16 [-0.04, 0.36]	16	0.30	0.13	
	**Subtotal**	**6**	**0.05 [-0.15, 0.24]**	**36**	**0.17**	**0.64**	**0.08**
5. Baseline TC							
> 5.17 mmol/L	Delphinidin-based	9	-0.40 [-0.83, 0.03]	90	< 0.00001	0.07	
	Malvidin-based	2	0.24 [0.03, 0.44]	0	0.96	0.02	
	**Subtotal**	**12**	**-0.28 [-0.63, 0.07]**	**90**	**< 0.00001**	**0.11**	**0.009**
≤ 5.17 mmol/L	Cyanidin-based	3	-0.16 [-0.57, 0.24]	20	0.29	0.43	
	Delphinidin-based	3	-0.02 [-0.28, 0.25]	0	0.75	0.89	
	Malvidin-based	2	-0.09 [-0.42, 0.25]	0	0.77	0.61	
	**Subtotal**	**8**	**-0.09 [-0.26, 0.09]**	**0**	**0.80**	**0.34**	**0.83**
6. Baseline BMI							
≥ 25.0 kg/m^2	Cyanidin-based	3	-0.13 [-0.51, 0.24]	0	0.38	0.48	
	Delphinidin-based	6	-0.16 [-0.43, 0.12]	56	0.05	0.26	
	Malvidin-based	3	0.21 [0.02, 0.40]	0	0.75	0.03	
	**Subtotal**	**12**	**-0.04 [-0.21, 0.14]**	**47**	**0.04**	**0.70**	**0.06**
< 25.0 kg/m^2	Delphinidin-based	6	-0.14 [-0.32, 0.05]	0	0.96	0.14	
	**Subtotal**	**8**	**-0.17 [-0.32, 0.01]**	**0**	**0.94**	**0.03**	**0.56**

The probabilities are based on the Cochran's Q-test (a), test for overall effect of each anthocyanin group (b), and test for subgroup differences (c). If there was the data about subgroup numbers being one or less, analysis limited to that subgroup was omitted. BMI, body mass index; CI, confidence interval; MD, mean difference; n, sample size; TC, total cholesterol.
