# Supplementary material for: Differences in the Effects of Anthocyanin Supplementation on Glucose and Lipid Metabolism According to the Structure of the Main Anthocyanin: A Meta-Analysis of Randomized Controlled Trials
Source: Nutrients. 2021 Jun 10;13(6):2003. doi: 10.3390/nu13062003 (PMC8230537; doi:10.3390/nu13062003)
Supplement: Supplementary file 1 [file nutrients-13-02003-s001.zip › Table S3.pdf]

**Table S3.** Subgroup analyses for effects of anthocyanins and main anthocyanin in the test foods on LDL-C

| Subgroup | Anthocyanin groups | n | MD [95% CI] | $I^2$ | *p* Heterogeneity [a] | *p* Within-group [b] | *p* Intra-group [c] |
|---|---|---|---|---|---|---|---|
| 1. Main anthocyanin to total anthocyanin content | | | | | | | |
| ≥ 50% | Cyanidin-based | 2 | -0.24 [-0.58, 0.11] | 13 | 0.28 | 0.18 | |
| | Delphinidin-based | 8 | -0.24 [-0.34, -0.14] | 0 | 0.64 | < 0.00001 | |
| | **Subtotal** | **10** | **-0.24 [-0.33, -0.14]** | **0** | **0.71** | **< 0.00001** | **0.99** |
| < 50% | Delphinidin-based | 2 | -0.72 [-1.38, -0.04] | 80 | 0.02 | 0.03 | |
| | Malvidin-based | 4 | 0.05 [-0.12, 0.22] | 0 | 0.59 | 0.58 | |
| | **Subtotal** | **7** | **-0.19 [-0.50, 0.13]** | **77** | **0.0002** | **0.24** | **0.09** |
| 2. Anthocyanin dosage | | | | | | | |
| ≥ 160 mg | Delphinidin-based | 6 | -0.26 [-0.37, -0.16] | 0 | 0.63 | < 0.00001 | |
| | Malvidin-based | 2 | -0.09 [-0.41, 0.23] | 0 | 0.35 | 0.60 | |
| | **Subtotal** | **8** | **-0.25 [-0.35, -0.15]** | **0** | **0.62** | **< 0.00001** | **0.31** |
| < 160 mg | Cyanidin-based | 3 | -0.20 [-0.48, 0.09] | 0 | 0.46 | 0.17 | |
| | Delphinidin-based | 4 | -0.39 [-0.85, 0.07] | 79 | 0.002 | 0.10 | |
| | Malvidin-based | 2 | 0.10 [-0.10, 0.30] | 0 | 0.73 | 0.33 | |
| | **Subtotal** | **9** | **-0.19 [-0.43, 0.06]** | **70** | **0.0007** | **0.13** | **0.07** |
| 3. Types of anthocyanin source | | | | | | | |
| Purified anthocyanins | Delphinidin-based | 7 | -0.25 [-0.35, -0.15] | 0 | 0.63 | < 0.00001 | |
| | **Subtotal** | **7** | **-0.25 [-0.35, -0.15]** | **0** | **0.63** | **< 0.00001** | **-** |
| Extract | Cyanidin-based | 3 | -0.20 [-0.48, 0.09] | 0 | 0.46 | 0.17 | |
| | Delphinidin-based | 3 | -0.49 [-1.09, 0.10] | 81 | 0.006 | 0.10 | |
| | Malvidin-based | 3 | 0.04 [-0.14, 0.22] | 0 | 0.40 | 0.66 | |
| | **Subtotal** | **9** | **-0.20 [-0.45, 0.05]** | **70** | **0.0007** | **0.11** | **0.12** |
| 4. Target population | | | | | | | |
| Prediabetes and/or type 2 diabetes | Delphinidin-based | 2 | -0.23 [-0.41, -0.06] | 0 | 0.65 | 0.009 | |
| | **Subtotal** | **3** | **-0.19 [-0.39, 0.01]** | **0** | **0.43** | **0.02** | **0.22** |
| Dyslipidemia | Delphinidin-based | 6 | -0.36 [-0.60, -0.11] | 66 | 0.01 | 0.005 | |
| | **Subtotal** | **6** | **-0.36 [-0.60, -0.11]** | **66** | **0.01** | **0.005** | **-** |
| Overweight or obesity | Cyanidin-based | 2 | -0.24 [-0.58, 0.11] | 0 | 0.28 | 0.18 | |
| | **Subtotal** | **3** | **-0.18 [-0.45, 0.09]** | **0** | **0.42** | **0.20** | **0.46** |
| Healthy | Malvidin-based | 3 | 0.04 [-0.14, 0.22] | 0 | 0.40 | 0.66 | |
| | **Subtotal** | **3** | **0.04 [-0.14, 0.22]** | **0** | **0.40** | **0.66** | **-** |

| | | | | | | | |
|---|---|---|---|---|---|---|---|
| 5. Baseline LDL-C | | | | | | | |
| > 3.6 mmol/L | Delphinidin-based | 4 | -0.35 [-0.81, 0.11] | 80 | 0.002 | 0.13 | |
| | **Subtotal** | **4** | **-0.35 [-0.81, 0.11]** | **80** | **0.002** | **0.13** | **-** |
| ≤ 3.6 mmol/L | Cyanidin-based | 3 | -0.20 [-0.48, 0.09] | 0 | 0.46 | 0.17 | |
| | Delphinidin-based | 6 | -0.27 [-0.38, 0.17] | 0 | 0.59 | < 0.00001 | |
| | Malvidin-based | 4 | 0.05 [-0.12, 0.22] | 0 | 0.59 | 0.58 | |
| | **Subtotal** | **13** | **-0.16 [-0.27, -0.05]** | **30** | **0.14** | **0.005** | **0.007** |
| 6. Baseline BMI | | | | | | | |
| ≥ 25.0 kg/m$^2$ | Cyanidin-based | 3 | -0.20 [-0.48, 0.09] | 0 | 0.46 | 0.17 | |
| | Delphinidin-based | 4 | -0.29 [-0.43, -0.14] | 0 | 0.36 | < 0.0001 | |
| | Malvidin-based | 3 | 0.10 [-0.08, 0.29] | 0 | 0.93 | 0.28 | |
| | **Subtotal** | **10** | **-0.12 [-0.27, 0.04]** | **46** | **0.06** | **0.14** | **0.005** |
| < 25.0 kg/m$^2$ | Delphinidin-based | 5 | -0.19 [-0.33, -0.05] | 0 | 0.86 | 0.006 | |
| | **Subtotal** | **6** | **-0.19 [-0.51, -0.07]** | **0** | **0.93** | **0.004** | **0.98** |

The probabilities are based on the Cochran's Q-test (a), test for overall effect of each anthocyanin group (b), and test for subgroup differences (c). If there was the data about subgroup numbers being one or less, analysis limited to that subgroup was omitted. BMI, body mass index; CI, confidence interval; MD, mean difference; n, sample size; LDL-C, low-density lipoprotein cholesterol.
