# Supplementary material for: Differences in the Effects of Anthocyanin Supplementation on Glucose and Lipid Metabolism According to the Structure of the Main Anthocyanin: A Meta-Analysis of Randomized Controlled Trials
Source: Nutrients. 2021 Jun 10;13(6):2003. doi: 10.3390/nu13062003 (PMC8230537; doi:10.3390/nu13062003)
Supplement: Supplementary file 1 [file nutrients-13-02003-s001.zip › Table S6.pdf]

Table S6. Subgroup analyses for effects of anthocyanins and main anthocyanin in the test foods on Insulin

Subgroup	Anthocyanin groups	n	MD [95% CI]	I^2	*p*		
					Heterogeneity [a]	Within-group [b]	Intra-group [c]
1. Main anthocyanin to total anthocyanin content							
≥ 50%	Cyanidin-based	2	0.58 [-3.95, 5.12]	71	0.07	0.80	
	Delphinidin-based	3	-0.32 [-0.91, 0.28]	0	0.85	0.30	
	**Subtotal**	**5**	**-0.30 [-0.89, 0.29]**	**0**	**0.42**	**0.32**	**0.70**
< 50%	**Subtotal**	**3**	**0.33 [-3.23, 3.90]**	**0**	**0.85**	**0.45**	**0.85**
2. Anthocyanin dosage							
≥ 160 mg	Delphinidin-based	4	-0.34 [-0.93, 0.26]	0	0.88	0.27	
	Subtotal	6	-0.28 [-0.87, 0.31]	3	0.40	0.35	0.11
< 160 mg	Cyanidin-based	2	-0.33 [-4.11, 3.44]	0	0.50	0.86	
	**Subtotal**	**2**	**-0.33 [-4.11, 3.44]**	**0**	**0.50**	**0.86**	**-**
3. Types of anthocyanin source							
Purified anthocyanins	Delphinidin-based	3	-0.32 [-0.91, 0.28]	0	0.85	0.30	
	**Subtotal**	**3**	**-0.32 [-0.91, 0.28]**	**0**	**0.85**	**0.30**	**-**
Extract	Cyanidin-based	3	0.80 [-2.75, 4.35]	42	0.18	0.66	
	**Subtotal**	**4**	**-0.04 [-3.01, 2.93]**	**27**	**0.25**	**0.98**	**0.40**
4. Target population							
Prediabetes and/or type 2 diabetes	Delphinidin-based	2	-0.59 [-2.03, 0.85]	0	0.69	0.42	
	Subtotal	3	-0.46 [-1.87, 0.96]	0	0.51	0.53	0.27
Metabolic syndrome	Cyanidin-based	2	3.04 [-1.96, 8.05]	46	0.17	0.23	
	**Subtotal**	**2**	**3.04 [-1.96, 8.05]**	**46**	**0.17**	**0.23**	**-**
Overweight or obesity	Cyanidin-based	1	-1.48 [-6.52, 3.56]	-	-	0.56	
	Subtotal	1	-1.48 [-6.52, 3.56]	-	-	0.56	-
Others	Delphinidin-based	2	-0.29 [-0.94, 0.37]	0	0.53	0.39	
	**Subtotal**	**2**	**-0.29 [-0.94, 0.37]**	**0**	**0.53**	**0.39**	**-**
5. Baseline Insulin							
> 11 μIU/mL	Delphinidin-based	3	-0.68 [-2.07, 0.71]	0	0.82	0.34	
	**Subtotal**	**5**	**-0.62 [-1.94, 0.71]**	**0**	**0.78**	**0.36**	**0.50**
≤ 11 μIU/mL	Cyanidin-based	2	3.04 [-1.96, 8.05]	46	0.17	0.23	
	**Subtotal**	**3**	**-0.20 [-0.86, 0.45]**	**43**	**0.17**	**0.54**	**0.20**
6. Baseline BMI							
≥ 25.0 kg/m^2	Cyanidin-based	3	0.80 [-2.75, 4.35]	42	0.18	0.66	

	**Subtotal**	**5**	**-0.20 [-0.84, 0.44]**	**17**	**0.31**	**0.54**	**0.50**
< 25.0 kg/m^2	Delphinidin-based	2	-0.59 [-2.03, 0.85]	0	0.69	0.42	
	**Subtotal**	**2**	**-0.59 [-2.03, 0.85]**	**0**	**0.69**	**0.42**	**-**

The probabilities are based on the Cochran's Q-test (a), test for overall effect of each anthocyanin group (b), and test for subgroup differences (c). If there was the data about subgroup numbers being one or less, analysis limited to that subgroup was omitted. BMI, body mass index; CI, confidence interval; MD, mean difference; n, sample size.
