# Supplementary material for: Differences in the Effects of Anthocyanin Supplementation on Glucose and Lipid Metabolism According to the Structure of the Main Anthocyanin: A Meta-Analysis of Randomized Controlled Trials
Source: Nutrients. 2021 Jun 10;13(6):2003. doi: 10.3390/nu13062003 (PMC8230537; doi:10.3390/nu13062003)
Supplement: Supplementary file 1 [file nutrients-13-02003-s001.zip › Table S8.pdf]

Table S8. Subgroup analyses for effects of anthocyanins and main anthocyanin in the test foods on HbA1c

Subgroup		Anthocyanins	n	MD [95% CI]	I^2	P		
						heterogeneity [a]	Within-group [b]	Intra-group [c]
1. Main anthocyanin to total anthocyanin content								
	≥ 50%	Delphinidin-based	2	-0.13 [-0.31, 0.04]	0	0.87	0.14	
		**Subtotal**	**3**	**-0.13 [-0.31, 0.05]**	**0**	**0.85**	**0.15**	**0.58**
2. Anthocyanin dosage								
	≥ 160 mg	Delphinidin-based	2	-0.13 [-0.31, 0.04]	0	0.87	0.14	
		**Subtotal**	**4**	**-0.14 [-0.30, 0.03]**	**0**	**0.94**	**0.11**	**0.83**
3. Types of anthocyanin source								
	Purified anthocyanins	Delphinidin-based	2	-0.13 [-0.31, 0.04]	0	0.87	0.14	
		**Subtotal**	**2**	**-0.13 [-0.31, 0.04]**	**0**	**0.87**	**0.14**	**-**
4. Target population								
	Prediabetes and/or type 2 diabetes	Delphinidin-based	2	-0.13 [-0.31, 0.04]	0	0.87	0.14	
		**Subtotal**	**3**	**-0.14 [-0.31, 0.03]**	**0**	**0.96**	**0.10**	**0.81**
5. Baseline HbA1c								
	> 7.0%	**Subtotal**	**2**	**-0.17 [-0.68, 0.34]**	**0**	**0.56**	**0.50**	**0.56**
	≤ 7.0%	Delphinidin-based	2	-0.13 [-0.31, 0.04]	0	0.87	0.14	
		**Subtotal**	**2**	**-0.13 [-0.31, 0.04]**	**0**	**0.87**	**0.14**	**-**
6. Baseline BMI								
	≥ 25.0 kg/m^2	**Subtotal**	**2**	**-0.17 [-0.68, 0.34]**	**0**	**0.56**	**0.50**	**0.56**
	< 25.0 kg/m^2	Delphinidin-based	2	-0.13 [-0.31, 0.04]	0	0.87	0.14	
		**Subtotal**	**2**	**-0.13 [-0.31, 0.04]**	**0**	**0.87**	**0.14**	**-**

The probabilities are based on the Cochran's Q-test (a), test for overall effect of each anthocyanin group (b), and test for subgroup differences (c). If there was the data about subgroup numbers being one or less, analysis limited to that subgroup was omitted. BMI, body mass index; CI, confidence interval; MD, mean difference; n, sample size; HbA1c, hemoglobin A1c.
